# Target Gene and Function Prediction of Differentially Expressed MicroRNAs in Lactating Mammary Glands of Dairy Goats

**DOI:** 10.1155/2013/917342

**Published:** 2013-09-30

**Authors:** Fei Dong, Zhi-Bin Ji, Cun-Xian Chen, Gui-Zhi Wang, Jian-Min Wang

**Affiliations:** College of Animal Science and Veterinary Medicine, Shandong Agricultural University, Daizong Road No. 61, Taian, Shandong 271018, China

## Abstract

MicroRNAs are small noncoding RNAs that can regulate gene expression, and they can be involved in the regulation of mammary gland development. The differential expression of miRNAs during mammary gland development is expected to provide insight into their roles in regulating the homeostasis of mammary gland tissues. To screen out miRNAs that should have important regulatory function in the development of mammary gland from miRNA expression profiles and to predict their function, in this study, the target genes of differentially expressed miRNAs in the lactating mammary glands of Laoshan dairy goats are predicted, and then the functions of these miRNAs are analyzed via bioinformatics. First, we screen the expression patterns of 25 miRNAs that had shown significant differences during the different lactation stages in the mammary gland. Then, these miRNAs are clustered according to their expression patterns. Computational methods were used to obtain 215 target genes for 22 of these miRNAs. Combining gene ontology annotation, Fisher's exact test, and KEGG analysis with the target prediction for these miRNAs, the regulatory functions of miRNAs belonging to different clusters are predicted.

## 1. Introduction

MicroRNAs (miRNAs) are endogenous ~22 nt RNAs that play an important role in regulating gene expression through sequence-specific base pairing with target mRNAs in animals and plants [1]. In animal cells, most studied miRNAs are formed into imperfect hybrids with sequences in the mRNA 3′-untranslated region (3′-UTR) and regulate cell development, cell proliferation, cell death, and morphogenesis [2, 3]. The key to understanding the miRNA regulatory mechanism is the ability to identify their regulatory targets. Computational prediction methods have developed into important approaches for obtaining these regulatory targets [4–6]. In plants, many miRNA targets can be predicted with confidence simply by searching for mRNAs with extensive complementarity to the miRNAs [7]. However in animals, miRNA target prediction is more difficult because of the incomplete complementary of the miRNA with its target, leading to many false predictions [4, 8]. TargetScan predicts biological targets of miRNAs by searching for the presence of conserved 8mer and 7mer sites that match the seed region of each miRNA [9]. PITA can predict miRNA targets in consideration of mRNA secondary structure [10]. miRGen is an integrated database that contains animal miRNA targets according to combinations of six target prediction programs. The mammary gland undergoes cycles of cell division, differentiation, and dedifferentiation in the adult ruminant [11], which is called lactation cycle. The Laoshan dairy goat, one of the most outstanding dairy goat breeds in China, is an ideal lactation research model for studying the molecular mechanisms of mammary gland development and lactating. miRNAs that demonstrate importance for development, cell proliferation, cell death, and morphogenesis should be involved in the regulation of the mammary gland. Many studies have shown that miRNAs influence mammary gland development by affecting the posttranscriptional expression of their target genes [12–14]. Classifying the function of these miRNA target genes, clustering that combines the expression patterns of the miRNAs will help construct a better understanding of the role of miRNAs in mammary gland tissues. With respect to comparative analyses of the function of the target genes (whether cross-species or cross-library), systematic annotation descriptors are very powerful. Gene ontology (GO) provides a controlled vocabulary to describe gene products [15]. The Kyoto Encyclopedia for Genes and Genomes (KEGG) provides the annotation of protein interaction networks (PATHWAY database) and chemical reactions (LIGAND database) that are responsible for various cellular processes [16].

With the development of next generation sequencing, a lot of miRNAs in different species and different tissues have been identified. However, a method that is able to screen out the miRNAs with vital regulatory function from numerous normal miRNAs is still needed. The goat is an ideal lactation research model for studying the molecular mechanisms of mammary gland development and lactating. Hence, the miRNAs that identified differentially expressed among goats could provide an insight to regulatory mechanism of lactation.

Our study is based on miRNA the expression profiles in the mammary gland of Laoshan dairy goats (*Capra hircus*) at different lactation stages (early lactation, peak lactation, and late lactation) that are obtained by Solexa deep-sequencing technology, which is considered to be a robust tool for miRNA identification on a large scale [17, 18]. First, we selected miRNAs that show significant differential expression at different stages. Then, the target genes for the selected miRNAs were predicted by several computational prediction algorithms. Next, Gene Ontology annotation, Fisher's exact test, and KEGG analysis of the target genes are performed by bioinformatics. Finally, combining the target prediction with bioinformatic analysis systematically, the regulatory roles of these miRNAs in the mammary gland during lactation period were predicted. This work could provide theoretical support and clues for the in-depth study of specific miRNA-target pairs in mammary gland tissue. In terms of analysis of miRNA deep sequencing data from other species and tissues, this method would be effective as well.

## 2. Materials and Methods

### 2.1. Screening and Cluster Analysis of miRNA Expression Profile

The miRNA expression profile of Laoshan dairy goat mammary glands at three different lactation stages (early lactation, peak lactation, and late lactation) is obtained through Solexa deep-sequencing technology. To obtain significant miRNA expression profiles, five healthy Laoshan dairy goats in three lactation period (four years old) were used. Total RNAs of mammary gland tissue of five goats were extracted by using TRIzol reagent (Invitrogen). The small RNA libraries were constructed by using the homogenized and pooled total RNAs of five goats from three lactation stages, respectively. Then these RNA libraries were constructed and sequenced by using an Illumina/Solexa 1G Genome Analyzer at the Beijing Genomics Institute (BGI), Shenzhen, China. The deep-sequencing data were verified to be high quality and reliable for the expression profiling analysis of miRNAs in our previous studies (see [19, 20]). Parts of the raw data have been deposited in the Gene Expression Omnibus (GEO) at NCBI with accession number GSE39484.

First, we compared the expression of miRNAs among the three lactation stages, and the expression levels of the miRNAs in three samples were normalized to obtain the transcript expression per million. In this study, the early and peak lactation stages expressed miRNAs with significant differences and with a normalized expression more than 50 transcript expression per million in at least one stage are selected for further analysis. The criterion for a significant difference is fold change (log⁡⁡2) > 1 or fold change (log⁡⁡2)<−1 with *P* value < 0.01. The fold change and *P* value were calculated from the normalized expression data using the following formulas.

(1) Normalized  expression  =(actual  miRNA  sequencing  reads  counttotal  clean  reads  count×1,000,000),

fold change = Log 2(*H*/*S*).

*P* value:

(2) p(x ∣ y)=  (N1N2)y ×(x+y)!x!y!(1+(N2/N1))(x+y+1)C(y≤ymin⁡ ∣ x)=∑y=0y≤ymin⁡p(y ∣ x)D(y≥ymax⁡ ∣ x)=∑y≥ymax⁡∞p(y ∣ x),

where *N*_1_ and *x* represent the total counts of clean reads and normalized expression, respectively, for a given miRNA in the peak lactation sRNA library, and *N*_2_ and *y* represent the total counts of clean reads and normalized expression, respectively, for a given miRNA in the late lactation sRNA library.

Then, the selected miRNAs are clustered according to their expression abundance in the three stages. The clustering dendrogram of the miRNAs is drawn using IBM SPSS statistic version 19 software (IBM SPSS Statistics Inc., Chicago, IL, USA) by hierarchical cluster analysis based on between-group linkage.

### 2.2. Prediction and Screening of miRNA Target Genes

The target genes of the selected miRNAs are predicted using eight prediction algorithms due to the potential of target prediction results with a high false-positive rate in animals. The tools include TargetScanHuman 6.2 [9] (http://www.targetscan.org/) and PITA [10] (http://genie.weizmann.ac.il/pubs/mir07/). One database, miRGen v3 [21] is an integrated database that includes the prediction results of six algorithms, DIANA-microT, miRanda (microran.org), miRanda (miRBase), PicTar (4-way), PicTar (5-way), and TargetScanS. In miRGen, all PicTar targets were downloaded as UCSC Tracks from the UCSC Genome Browser Database. “4-way” refers to a 4-species conservation constraint (human/mouse/rat/dog), and “5-way” refers to a 5-species conservation constraint (human/mouse/rat/dog/chicken). Human targets predicted by TargetScanS were downloaded from the TargetScan Release 3.1 download site. All miRanda “miRBase” targets were downloaded from the miRBase Targets Release Version 4. All miRanda “microrna.org” targets were downloaded from the website http://www.microrna.org/. All DIANA-microT targets were obtained directly from the DIANA lab. In TargetScan, we select cow as the predicted species, and, in the other programs, we select* Homo sapiens *as the predicted species. In PITA, we set the minimum seed size as 7, allow a single mismatch, and use the default settings for all other parameters. Then, the target genes that represent the intersection of at least 3 algorithms are selected as candidate target genes for further analysis.

### 2.3. Bioinformatic Analysis of Candidate Target Genes

The nucleotide sequences of the candidate target genes are downloaded from the NCBI nucleotide database. Here,* Bos taurus* gene sequences are downloaded because the study of *Capra hircus* genes is deficient. Then these sequence data are loaded into Blast2GO software which is a comprehensive bioinformatics tool for the functional annotation and analysis of gene or protein sequences [22]. GO annotation, EC annotation, and KEGG maps of candidate target genes are obtained through Blast2GO. Then, the DAVID Bioinformatics Resources 6.7 (http://david.abcc.ncifcrf.gov/home.jsp) is used to make KEGG pathway annotations [23, 24]. Finally, GO enrichment analysis and Fisher's exact tests of these genes are performed to reveal the relationships between the miRNAs and their possible regulatory functions.

The score of the GO enrichment at each node is computed according to the formula:

(3) $$\begin{array}{c} \text{score}=\sum_{\text{GOs}}\text{seq}\times \alpha^{\mathrm{dist}}, \end{array}$$

where seq is the number of different sequences annotated at a child GO term and dist is the distance to the node of the child.

Fisher's exact test of the target genes of different miRNA clusters is based on the following rules: set GO terms of the target genes of different miRNA clusters as the test set and the others as the reference set; set *P* < 0.05 as the limit of significance.

To make KEGG pathway annotation, first we submit target gene set to function annotation tool of DAVID Bioinformatics Resources. Then *Bos taurus* is selected as background. At last, we select KEGG pathway in pathway and obtained function annotation table of KEGG pathway.

## 3. Results and Discussion

### 3.1. Screening and Cluster Analysis of miRNA Expression Profiles

**Figure 1:**
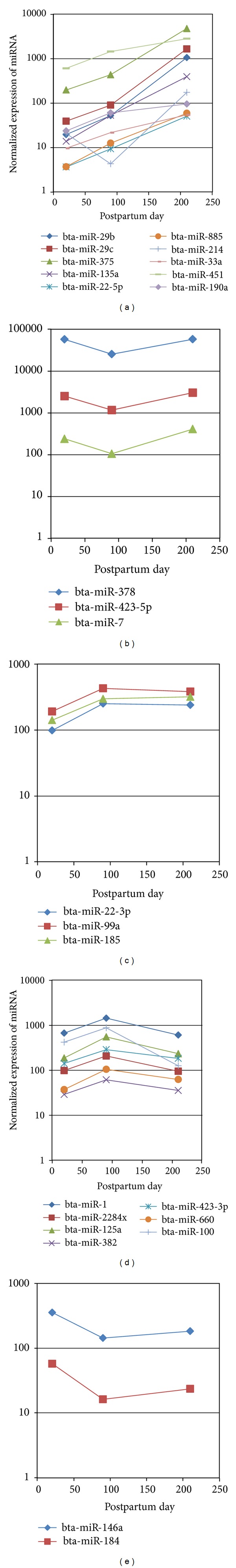
The expression patterns of 25 miRNAs belonging to five classifications. These miRNAs are clustered according to their expressions at three stages by SPSS 19.0. The three stages are early lactation (20 days postpartum), peak lactation (90 days postpartum), and late lactation (210 days postpartum). (a) The expression patterns of miRNAs that increase during these three stages. (b) The expression patterns of miRNAs that showed the lowest expression level at peak lactation. (c) The expression of miRNAs that showed a similar level at peak and late lactation but were higher than at early lactation. (d) The expression patterns of miRNAs that showed the highest level at peak lactation. (e) The expression patterns of miRNAs that showed a similar level at peak and late lactation but were lower than at early lactation.

The dairy goat lactation cycle usually is divided into four stages: early lactation, peak lactation, late lactation, and dry period. The numbers and secretory activity of mammary epithelial cells significantly increased from early lactation to peak lactation. After peak lactation, the apoptosis in mammary epithelial cells started. The milk yield and composition are directly influenced by the numbers and secretory activity of mammary epithelial cells. The expression levels of some miRNAs change significantly during different development stages, suggesting that they likely have a regulatory function in mammary gland development. Initially, 56 miRNAs that are significantly over or underexpressed are selected by comparing peak lactation (90 days postpartum) with early lactation (20 days postpartum). Then, the miRNAs that express less than 50 at all three stages are deleted because the results show that the average normalized expression of all miRNAs in the three samples is greater than 2500. Here, 25 miRNAs are selected for follow-up analysis. Next, these miRNAs are clustered according to their expression at three stages by SPSS 19.0. The result shows that these miRNAs fall into five classes (cluster A–E). The expression patterns of the different clustered miRNAs are shown in Figure 1. The miRNAs are named as bta-miR-XXX because the raw data were matched with the known *Bos taurus* miRNAs in miRBase 17.0.

Some predicted miRNAs have propensity to target genes with related functions [25]. Studies have also shown that the expression of miRNA target genes is significantly correlated with the expression of their miRNAs [26]. Here, we hypothesize that miRNAs with very striking expression specificities belong to different classes that likely have highly similar specific functions or roles. The expression patterns of miRNAs in the same cluster are similar, which suggests that these miRNAs are highly coregulating miRNAs in mammary gland. Although the regulatory role of miRNAs likely contains “switch,” “tuning,” and “neutral” effects [4], a recent study showed that most mammalian miRNAs predominantly act to repress the expression of their target genes [27]. The expression patterns of miRNAs belonging to same cluster are mostly similar, but miR-214 in group A differs from the others of group A as its expression is lowest at peak lactation. These miRNAs are clustered according to the hierarchical clustering method by SPSS 19.0, and this method is calculated based on the relative distance of each component. The normalized expressions of miR-214 at three stages, respectively, are 20.7968, 4.3058, and 173.9817, respectively. Since the expressions at early and peak lactation are very low, we still included miR-214 in group A. To reveal the correlations of the miRNAs in these five classes, we then analyzed the target genes of these miRNAs that are obtained through target prediction.

### 3.2. Prediction and Screening of miRNA Target Genes

The function of a miRNA is ultimately defined by the genes that it targets and by its effect on the expression of those genes. In animals, miRNA target prediction is more difficult than that in plants because of the incomplete complementarity. To encounter this problem, we used eight methods and databases to predict the miRNA target genes. During the target prediction process, we select cow as the predicted species in TargetScanHuman 6.2 and select human as the predicted species in the remaining programs. The main reasons for these selections are the following: (1) the prediction of *Capra hircus* target genes is not available in these programs, and the genetic relationship between cow and goat is very close; (2) the mammalian target sites of miRNAs are usually conserved given that the miRNAs are highly evolutionarily conserved [28], and many algorithms are primarily developed for *Homo sapiens*. The target prediction results of different programs are indicated in Table 1.

Among these eight methods, the PITA software obtained the most target genes as the predicted targets contain all the genes that paired to 7-8 “seed regions” with a low degree of conservation and no restriction for the ΔΔ*G* score, which is the energetic score of PITA. Meanwhile, the number of targets obtained by DIANA-microT is lowest, because the algorithm of DIANA-microT uses up to 27 species to access the “seed region” conservation profile with an additional comparative step [29]. It should be noted that the results of the DIANA-microT prediction, which were obtained through miRGen, are lower than those of the original DIANA-microT (http://diana.cslab.ece.ntua.gr/microT/). All of these different algorithms have the same basic criteria for predicting target genes, such as the complementarity between the miRNA “seed region” and the target sites, the conservation of miRNA target sites among different species, the thermal stability of the miRNA/mRNA duplex, and the absence of complicated secondary structures in miRNA target sites [8]. Subsequently, different programs have further increased or improved the constraints, and we can obtain more reliable results. For example, TargetScan rewards an A across from position 1, whereas the other algorithms with stringent seed pairing reward a Watson-Crick match. Although these methods have predictive value, they presented a lower rate of success in predicting the responsive targets when we included the experimental verification [30]. As more programs are used as references, the possibility of predicting miRNA target genes should be more reliable.

In this case, we select target genes that represent the intersection of at least three programs to reduce the false-positive rate.  Table 2 demonstrates that the candidate target genes of the miRNAs possess a high probability of being true positives. According to the prediction under restrictive conditions, a total of 215 target genes for 22 miRNAs are obtained. The candidate target genes for three miRNAs (bta-miR-885, bta-miR-423-3p, and bta-miR-2284x) cannot be obtained because most of the programs are unable to include them. One particular gene, *STAB2,* is regulated by 3 of 22 miRNAs, and, at the same time, 36 genes are regulated by 2 miRNAs. Some predicted target genes have been validated. For example, several researches have shown that miR-29b negatively regulates the expression of several collagen genes that contain *COL3A1 *[31, 32]. Garzon et al. showed that expression of miR-29b in acute myeloid leukemia cells resulted in marked reduction of the expression of DNA methyltransferases DNMT3A at both RNA and protein levels [33]. And YAP1 is validated suppressed by mir-375 [34, 35].

### 3.3. Bioinformatics Analysis of Candidate Target Genes

#### 3.3.1. Gene Ontology Annotation of Target Genes

**Figure 2:**
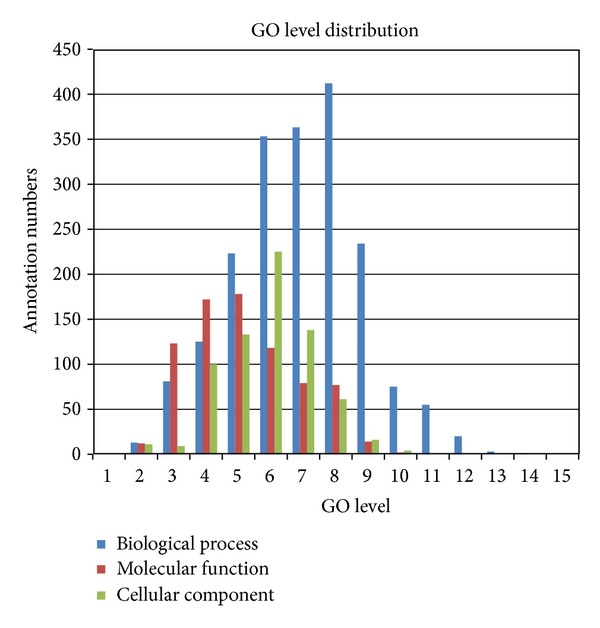
The annotated GO level distributions of three GO domains.

To identify the actual regulatory functions of the miRNAs, the GO annotation of the target genes was performed. The GO project is a bioinformatic resource providing functional information about gene products and describing functions through the adoption of domain-specific ontologies [15]. Additionally, the GO information is more suitable for the functional analysis of miRNAs. GO annotation is based on the protein expression level. Furthermore, some miRNAs inhibit protein production but do not change the mRNA expression levels [36]. The ontology covers three domains: cellular component, molecular function, and biological process. In addition, the GO information is structured as a directed acyclic graph, and each term has defined relationships to one or more other terms in the same domain. In the course of our experiment, 212 homologous genes in *Bos taurus* are downloaded from NCBI, and three genes (*EBF3*,* C13orf23,* and* SGK*) are still not found in *Bos taurus*. Through annotation in Blast2GO, a total of 3436 GO terms for 211 genes were acquired, and one gene, *FNBP4,* was not annotated in the GO database. The GO terms of biological processes are mainly distributed in levels 6–8 and the GO terms of molecular function and cellular component are mainly distributed in levels 5 and 6 (shown in Figure 2). GO has a hierarchical structure starting with top-level ontologies for molecular functions, biological processes, and cellular components. With the annotation level increased, it can facilitate the extraction of more detailedfunctional information. 

**Figure 3:**
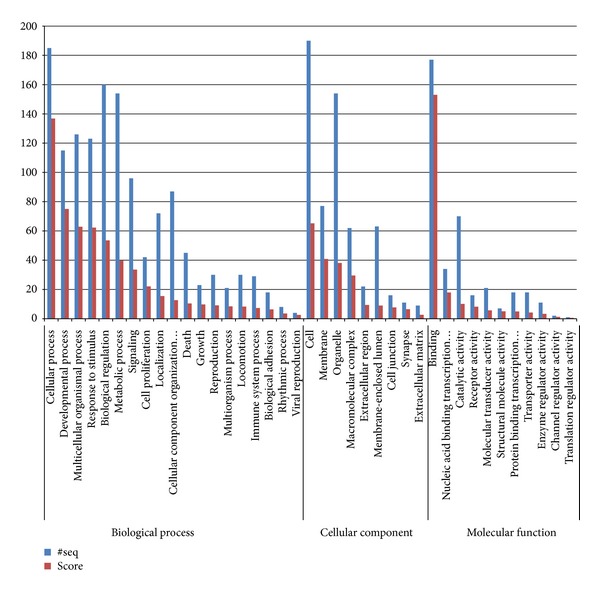
GO classification of the target genes at level 2 that were annotated through Blast2GO.

In our studies we find that level 2 typically maintains good coverage while also providing meaningful term specificity. The GO enrichment analysis of the three domains in GO level 2 shows that most sequences (185 sequences with a score of 136.79) participate in cellular processes in the biological process domain, most (177 sequences with a score of 152.98) have a broad binding function in the molecular function domain, and most are located in the cell (190 sequences with a score of 65.18), membrane (77 sequences with a score of 40.83), and organelle (154 sequences with a score of 38.13).  Figure 3 describes the GO classification of the target genes at level 2 (for the statistical of GO terms see additional file 1 in the Supplementary Material available online at http://dx.doi.org/10.1155/2013/917342). The ten most enriched GO terms in the three domains are shown in Table 3.

The most enriched GO terms of the target genes are mostly involved in developmental processes, showing the same conclusion as that by Farh et al. that genes related to developmental processes are enriched for miRNA sites [37]. At the same time, many terms are likely related to the physiological processes of the mammary gland. For example, multicellular organismal processes and ATP binding functions are related to mammary gland epithelial cell proliferation and apoptosis. Furthermore, protein binding and metal ion binding are closely related to milk secretion.

#### 3.3.2. The Enrichment Analysis of miRNAs Targets

**Figure 4:**
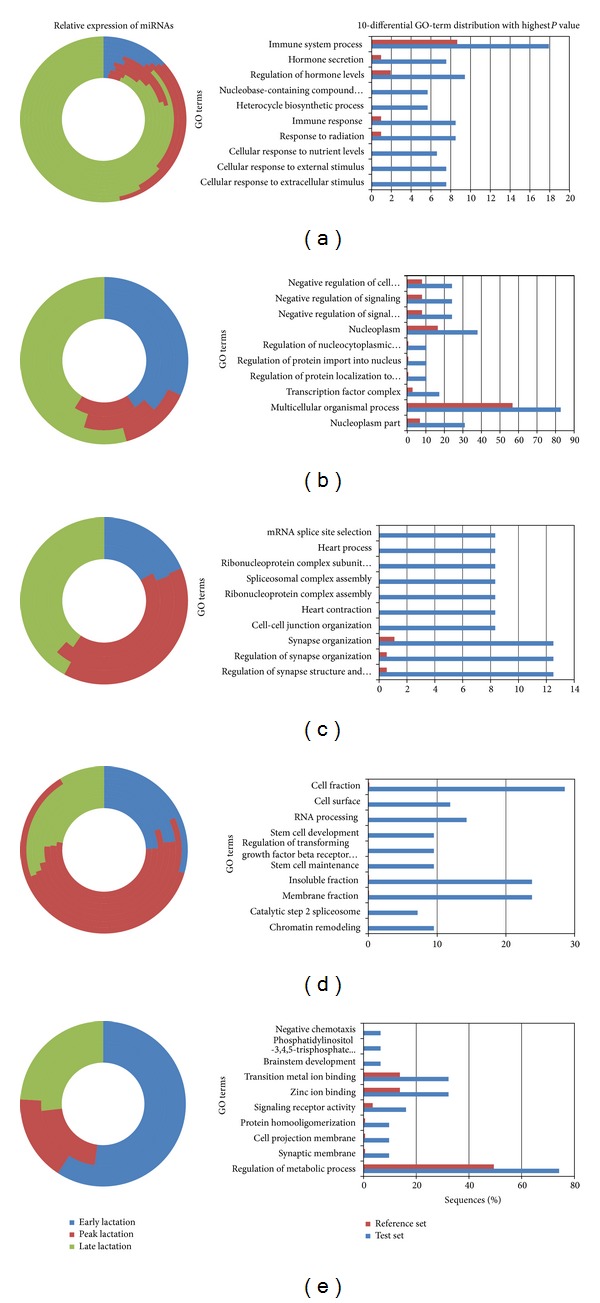
The expression pattern of miRNAs and the significantly enriched GO annotations of their target genes. The relative expression patterns of miRNAs among the three stages are shown on the left side, and the significantly enrichd GO terms of that cluster are shown on the right side. Every circle represents an miRNA expression pattern: (a) bta-miR-29b, bta-miR-29c, bta-miR-375, bta-miR-135a, bta-miR-22-5p, bta-miR-885, bta-miR-214, bta-miR-33a, bta-miR-451, and bta-miR-190a; (b) bta-miR-378, bta-miR-423-5p, and bta-miR-7; (c) bta-miR-22-3p, bta-miR-99a, and bta-miR-185; (d) bta-miR-1, bta-miR-2284x, bta-miR-125a, bta-miR-382, bta-miR-423-3p, bta-miR-660, and bta-miR-100; (e) bta-miR-146a and bta-miR-184. The order is given from inside to outside.

To test whether the functions, processes, or locations described in GO are significantly enriched in the clustered genes compared with the reference group, we conducted Fisher's exact test for the target genes of different clustered miRNAs [38]. Then, different GO annotations and miRNAs with different expressions are connected together. Thus, the significant GO terms of differentially expressed miRNAs are predicted (shown in Figure 4 and additional file 2). 

The GO terms we obtained in all test sets are all overannotated. Among the GO terms of the target genes for the miRNAs in cluster A, cellular response to nutrient levels and cellular response to extracellular stimulus (GO: 0009605) are overannotated. Among the genes annotated to this term,* VLDLR, PTK2, ADCYAP1, KCNMA1, and WAC *are all target genes of miR-135a. A research has shown that *VLDLR* expression was upregulated throughout lactation, particularly in the early lactation [39]. And their data suggest *VLDLR* play an important role in milk fat synthesis during lactation. GO terms such as insulin secretion, hormone secretion, peptide secretion, and peptide transport, which are all defined as transport (GO: 0006810), are related to transporting substances into, out of, or within a cell. Some cellular nitrogen compound metabolic processes are also overannotated. Some studies about these miRNAs have been conducted. Fang et al. showed that miR-29 plays an important role in the host antiviral defense through the activation of COX-2 and production of IFN-*λ*1 [40]. Rosenberger et al. reported that miR-451 regulates dendritic cell cytokine responses to influenza infection [41]. Beezhold et al. reported that elevated levels of miR-190 could downregulate the translation of PHLPP, which is a negative regulator of Akt signaling [42]. Combining these results with our own, we presume that the miRNAs of cluster A have regulatory functions in the response to stimuli, transport, and cellular nitrogen compound metabolic processes.

Most overannotated GO terms in cluster B are related to the regulation of protein transport (GO: 0051223), regulation of transport (GO: 0051049), regulation of histone acetylation (GO: 0035065), and regulation of locomotion (GO: 0040012). Giles et al. reported that insulin receptor substrate-2 (IRS-2) is a target of miR-7-5p and an activator of protein kinase B (Akt), which has important functions in glycometabolism and protein synthesis [43]. Estrogen is important for milk secretion, and Xu et al. reported that estradiol production is posttranscriptionally downregulated by miR-378 [44]. According to our study, the GO annotation shows that miR-378, miR-423-5p, and miR-7 (cluster B) should have important regulatory functions during lactation with respect to milk ingredient transport and milk ingredient synthesis in the mammary gland. Similarly, the miRNAs of cluster C are significantly enriched in the processes of cellular component organization, RNA splicing, and ribonucleoprotein complex assembly. The regulation of the DNA damage response, stem cell differentiation, and development are likely the regulatory functions of cluster D. In addition, miR-146a and miR-184 (cluster E) most likely participate in the regulation of phosphatase activity and the regulation of the insulin receptor signaling pathway.

Functionally related genes are usually regulated by a group of miRNAs instead of one miRNA. In this study, we clustered miRNAs based on their expression patterns as highly probable coregulated miRNAs and annotated the significant functions that may be regulated by these miRNAs. These significant GO annotations provide important guidance and clues for our follow-up study of specific miRNAs. However, there are still several drawbacks. If working with more genes, the Fisher's exact test will be more appropriate.

#### 3.3.3. Enzyme Code Annotation and KEGG Pathway

Kyoto Encyclopedia of Genes and Genomes (KEGG) is a database resource for understanding the high-level functions and utilities of biological systems. Its pathway-based analysis could facilitate the understanding of the biological functions of genes. KEGG pathways downloaded by the Blast2Go software are based on the enzyme code annotation of genes. There are 20 pathways associated with the *HADHB* gene, which shows that *HADHB* has important functions. The mitochondrial protein HADHB is required for the *β*-oxidation of fatty acids in mitochondria [45]. According to the target prediction, miR-33a could regulate *HADHB* to influence the *β*-oxidation rate of fatty acids in mammary gland cells.

To obtain more pathways related to these genes, we then used the DAVID Bioinformatics Resources to create KEGG pathway annotations. Totally 65 genes are annotated to 76 KEGG pathways. And the others are not annotated to KEGG. The pathways obtained are related to more than two genes, which are shown in Table 4. (The ID of genes is listed in additional file 3). Since the genes annotated to KEGG are so few, we could not cluster them to make enrichment analysis as GO enrichment analysis. Although we do not obtain pathways that are significantly enriched, the results indicate that miRNAs have important regulatory functions for cancer. Many genes that are predicted as miRNA candidate target genes take part in pathways related to cancer cell metastasis. 

In this study, most functional annotation and analysis of gene clusters are conducted through Blast2GO. The most important thing that needs to be done is screen and cluster miRNAs in line with detailed policy and rule. Once miRNAs are clustered clearly, the following step will be very easy. The function annotation of these miRNAs will help us find important miRNA for in-depth study. And this approach could apply to miRNA high-throughput sequencing data of other animals and tissues.

## 4. Conclusions

In this study, to identify important regulatory miRNAs in the goat mammary gland and to predict their regulatory functions, we constructed an effective and efficient procedure using bioinformatics to systematically analyze the biological functions of differentially expressed miRNAs that were acquired through high-throughput sequencing. A total of 215 target genes of 22 miRNAs are obtained, and the regulatory function of miRNAs with similar expression patterns is annotated through GO annotation, Fisher's exact test, and KEGG analysis. This procedure enabled the selection of miRNAs with important regulatory functions and provided clues and theoretical guidance for the study of these 22 specific miRNAs with important regulatory functions in mammary gland development.

According to the enriched GO annotation, miR-378, miR-423-5p, and miR-7 (cluster B) should have especially important regulatory functions in mammary gland biology during lactation regarding milk ingredient transport and ingredient synthesis.

## Supplementary Material

Additional file 1 contains all GO terms that are annotated to candidate target genes. The table contains all GO terms, ID, score and the number of genes be annotated to this term. The three sheets respectively are GO terms of biological process, cell component and molecular function.

## Figures and Tables

**Table 1:** The number of targets predicted by the eight prediction software.

miRNA name	TargetScan	DIANA-microT	miRanda (http://www.microrna.org/)	miRanda (miRBase)	PicTar (4-way)	PicTar (5-way)	TargetscanS	PITA
bta-miR-29b	962	15	993	304	527	145	602	941
bta-miR-29c	44	14	981	305	532	147	602	3344
bta-miR-375	646	1	787	0	159	34	180	717
bta-miR-135a	610	15	930	0	327	108	397	2279
bta-miR-22-5p	252	10	751	206	258	71	252	1409
bta-miR-885	184	0	0	0	0	0	0	0
bta-miR-214	394	10	844	318	401	103	327	1936
bta-miR-33a	372	11	883	211	246	103	208	1555
bta-miR-451	22	1	815	0	0	0	14	1971
bta-miR-190a	177	8	677	157	88	39	86	1696
bta-miR-378	151	3	623	0	0	0	284	3400
bta-miR-423-5p	159	0	722	0	0	0	9	6161
bta-miR-7	403	15	628	173	234	61	217	1793
bta-miR-423-3p	10	0	722	0	0	0	9	678
bta-miR-99a	18	0	772	9	33	22	32	309
bta-miR-185	281	17	753	281	248	0	162	4171
bta-miR-1	577	18	737	266	390	156	443	2146
bta-miR-2284x	618	0	0	0	0	0	0	0
bta-miR-125a	301	5	834	212	392	0	436	0
bta-miR-382	189	8	755	0	0	0	91	2431
bta-miR-22-3p	465	10	751	206	258	71	252	1409
bta-miR-660	132	0	521	0	0	0	0	615
bta-miR-100	29	1	692	31	32	22	32	309
bta-miR-146a	203	0	772	0	99	18	104	1858
bta-miR-184	25	1	775	87	19	2	16	1425

**Table 2:** Candidate target genes for differentially expressed miRNAs obtained by the intersection of at least three programs.

miRNA name	Target gene	TG^1^	NS^2^
bta-miR-29b	*COL3A1, FBN1, COL11A1, COL1A2, IREB2, TRIB2, MYCN, BLMH, PPM1D, DNMT3A, ISL1, PDGFB, ARVCF, ZBTB40 *	14	7
bta-miR-29c	*TRAF4, PMP22, COL4A2, DNAJB11, CRISPLD1, PDGFB, FBN1, BLMH, ISL1, YY1, PPM1D, IREB2, FOS, MYCN, RLF, HMCN1, HMGN3, DNMT3A, SGK *	19	6
bta-miR-375	*YBX1, QKI, ELAVL4, ZFPM2, HOXA3, UBE3A, PDE4D, YAP1, TSC22D2, TIMM8A, EBF3, PRKD1, HABP2, C13orf23 *	14	5
bta-miR-135a	*NR3C2, CTTNBP2, SP3, VLDLR, PTK2, ARHGEF4, ADCYAP1, KCNMA1, BTBD10, WAC, NBEA, INHBA, TRPM7, ELOVL6, RAP2A, USP15 *	16	6
bta-miR-22-5p	*BIN1, WDR26, SV2A, SATB2, BCL9, MAT2A, MAP3K12, FNBP4, FAM49B, BCL9L, YARS, RBM15, WDFY3, STAG2, DNAJB5, WDTC1, SIRT1, LRRC1 *	18	5
bta-miR-214	*ZBTB20, ARPC5L, KPNA3, ARVCF, RAB14, WDTC1, ITCH, KLC2, RTN2 *	9	6
bta-miR-33a	*ABCA1, KPNA4, ZNF281, SLC25A25, YWHAH, SATB2, HADHB, EEF1A1, CACNA1C, CAMK2G, ATP1B1 *	11	6
bta-miR-451	*TTN, OSR1, PMM2, C11orf30, AEBP2, SAMD4B, TBX1, FBXO33, CDKN2D, YWHAZ, YTHDF2, CAB39, ZNF644 *	13	3
bta-miR-190a	*NEUROD1, WSB1, TNRC6A, NBEA, HECA, C20o*r*f112 *	6	6
bta-miR-378	*TOB2, C11orf49, SOX7, DDAH1, SULF1, SRC, DYRK1A, DBT, STAC2 *	9	3
bta-miR-423-5p	*SUFU, NTN1, STK24, AP2A1, DAB2IP, MYBL2, CAMTA2 *	7	3
bta-miR-7	*SPATA2, ARID4A, GLI3, SNCA, KCNJ2, KLF4, WDR47, PDE4D, PFN2, GATA6, PHF21A, LEMD3, PPARGC1A *	13	6
bta-miR-99a	*HOXA1, MBNL1, MTMR3, HS3ST2, SMAD7, HNRPU *	6	5
bta-miR-185	*ATP6V1F, IKZF4, SLC16A2, CA10, EPHB2, PAK6, DTX3, NEUROD2, CDC42, EIF2C1, SF1, PHYHIP, CPEB2, FAM53B, PRX, RAB14, GATAD2B, IGSF4C, RAE1 *	19	5
bta-miR-1	*FNDC3A, E2F5, KTN1, DDX5, CLTC, EIF4E *	6	7
bta-miR-125a	*GTPBP2, RAPGEFL1, ABHD3, ATP1B4, PPP1CA, ACCN2 *	6	5
bta-miR-382	*TOP1, HSPA2, EEF1A1, RPE, SYNCRIP, CLTC *	6	4
bta-miR-22-3p	*FAM49B, SIRT1, DNAJB5, RBM15, BCL9L, STAG2, LRRC1, FNBP4, SATB2, BCL9, MAP3K12, SV2A, WDFY3 *	13	6
bta-miR-660	*DCBLD2, SDC1, DNAJA2 *	3	3
bta-miR-100	*HS3ST2, FGFR3, SMARCA5, EIF2C2, FZD8, TRIB2, SMAD7, MTMR3, HOXA1, HNRPU *	10	5
bta-miR-146a	*IRAK1, STRBP, NOVA1, KLF7, LRRC15, USP3, STC1, CASK, SH3GL2, ROBO1, PHOX2B, PPP1R11, RUNX1T1, ZFYVE1, BCL11A, CNTFR, MYT1, ELAVL1, SYT1, * *NRP2, HIC2, EIF5A2, FBXL10, KCTD15, DLGAP1, SLC25A14, SPIB *	27	4
bta-miR-184	*SF1, SIDT2, EIF2C2, CREB3L1, INPPL1, RASL10B, PRKCB1 *	7	4

^1^Total of genes that are selected as candidate target genes.

^2^The number of programs that can predict these candidate target genes.

**Table 3:** The ten most enriched GO terms of target genes on all levels in the three domains.

GO domain	GO ID	Term	TS^1^	Score	GO level
Biological_process	GO: 0008150	Biological process	195	180.95	1
	GO: 0009987	Cellular process	185	136.79	2
	GO: 0050794	Regulation of cellular process	150	98.57	4
	GO: 0050789	Regulation of biological process	157	79.43	3
	GO: 0032502	Developmental process	115	74.99	2
	GO: 0048856	Anatomical structure development	103	72.83	3
	GO: 0032501	Multicellular organismal process	126	62.86	2
	GO: 0007275	Multicellular organismal development	106	62.29	3
	GO: 0050896	Response to stimulus	123	62.25	2
	GO: 0048731	System development	85	55.98	4

Molecular_function	GO: 0005488	Binding	177	152.98	2
	GO: 0005515	Protein binding	149	148.21	3
	GO: 0003674	Molecular function	190	126.3	1
	GO: 0003677	DNA binding	51	40.89	4
	GO: 0003676	Nucleic acid binding	72	40.37	3
	GO: 0046872	Metal ion binding	59	36.02	5
	GO: 0008270	Zinc ion binding	34	34	7
	GO: 0003700	Sequence-specific DNA-binding transcription factor activity	33	28.16	3
	GO: 0005524	ATP binding	25	25	8
	GO: 0043169	Cation binding	59	21.61	4

Cellular_component	GO: 0044464	Cell part	190	105.3	3
	GO: 0005737	Cytoplasm	129	90.45	5
	GO: 0044424	Intracellular part	171	87.68	4
	GO: 0005575	Cellular component	201	82.84	1
	GO: 0044444	Cytoplasmic part	87	81.41	5
	GO: 0005634	Nucleus	111	78.92	7
	GO: 0043231	Intracellular membrane-bounded organelle	144	76.8	6
	GO: 0005623	Cell	190	65.18	2
	GO: 0043229	Intracellular organelle	154	62.49	5
	GO: 0005622	Intracellular	175	57.61	4

^1^The total of gene sequences that were annotated to this term.

**Table 4:** The KEGG pathways and annotated target genes.

Pathway ID	Pathway	Related gene
bta05200	Pathways in cancer	*GLI3, PTK2, TRAF4, CDC42, COL4A2, FGFR3, FZD8, RUNX1T1, PDGFB, SUFU, FOS *
bta04510	Focal adhesion	*PTK2, CDC42, COL1A2, COL3A1, COL4A2, COL11A1, PAK6, PPP1CA, PDGFB, SRC *
bta04810	Regulation of actin cytoskeleton	*PTK2, ARHGEF4, ARPC5L, CDC42, FGFR3, PAK6, PFN2, PPP1CA, PDGFB *
bta04360	Axon guidance	*EPHB2, PTK2, CDC42, NTN1, PAK6, ROBO1 *
bta04144	Endocytosis	*SH3GL2, CDC42, CLTC, FGFR3, ITCH, SRC *
bta04010	MAPK signaling pathway	*CACNA1C, CDC42, MAP3K12, PDGFB, FOS, FGFR3 *
bta04512	ECM-receptor interaction	*COL1A2, COL3A1, COL4A2, COL11A1, SV2A, SDC1 *
bta04110	Cell cycle	*E2F5, CDKN2D, STAG2, YWHAH, YWHAZ *
bta04722	Neurotrophin signaling pathway	*CAMK2G, CDC42, IRAK1, YWHAH, YWHAZ *
bta04012	ErbB signaling pathway	*PTK2, CAMK2G, PAK6, SRC *
bta04912	GnRH signaling pathway	*CACNA1C, CAMK2G, CDC42, SRC *
bta04114	Oocyte meiosis	*CAMK2G, PPP1CA, YWHAH, YWHAZ *
bta04260	Cardiac muscle contraction	*ATP1B4, ATP1B1, CACNA1C *
bta04350	TGF-beta signaling pathway	*E2F5, SMAD7, INHBA *
bta05217	Basal cell carcinoma	*GLI3, FZD8, SUFU *
bta04370	VEGF signaling pathway	*PTK2, CDC42, SRC *
bta05222	Small cell lung cancer	*PTK2, TRAF4, COL4A2 *
bta04916	Melanogenesis	*CREB3L1, CAMK2G, FZD8 *
bta05016	Huntington's disease	*CREB3L1, CLTC, PPARGC1A *
bta04270	Vascular smooth muscle contraction	*CACNA1C, KCNMA1, PPP1CA *
bta04720	Long-term potentiation	*CACNA1C, CAMK2G, PPP1CA *
bta04530	Tight junction	*CASK, CDC42, SRC *
bta04660	T cell receptor signaling pathway	*CDC42, PAK6, FOS *
bta04910	Insulin signaling pathway	*EIF4E, PPARGC1A, PPP1CA *
